# Loss of lamin B1 is a biomarker to quantify cellular senescence in photoaged skin

**DOI:** 10.1038/s41598-017-15901-9

**Published:** 2017-11-15

**Authors:** Audrey Shimei Wang, Peh Fern Ong, Alexandre Chojnowski, Carlos Clavel, Oliver Dreesen

**Affiliations:** 1 Cell Ageing, Institute of Medical Biology, 8A Biomedical Grove, #06-06, Immunos, 138648 Singapore; 2 Developmental and Regenerative Biology, 8A Biomedical Grove, #06-06, Immunos, 138648 Singapore; 3 Hair & Pigment Development, Institute of Medical Biology, 8A Biomedical Grove, #06-06, Immunos, 138648 Singapore

## Abstract

Skin ageing is an inevitable consequence of life and accelerated by exposure to ultraviolet (UV) rays. Senescence is an irreversible growth arrest and senescent cells accumulate in ageing tissues, at sites of age-related pathologies and in pre-neoplastic lesions. Conventionally, senescent cells have been detected by senescence associated-β-galactosidase (SA-β-gal) staining, a procedure that requires enzymatic activity, which is lost in fixed tissue samples. We previously demonstrated that loss of lamin B1 is a novel marker to identify senescent cells. Here, we demonstrate that loss of lamin B1 facilitates the detection and quantification of senescent cells upon UV-exposure *in vitro* and upon chronic UV-exposure and skin regeneration *in vivo*. Taken together, this marker enables the study of environmental conditions on tissue ageing and regeneration *in vivo*, serves as a diagnostic tool to distinguish senescent from proliferating cells in pre-neoplastic lesions, and facilitates investigating the role of senescent cells in various age-related pathologies.

## Introduction

Cellular senescence is an irreversible growth arrest and a fundamental process that determines cellular ageing and plays a critical role in tumor suppression^1–3^. Senescent cells accumulate with age in many tissues, including skin, in benign nevi, pre-neoplastic lesions and at sites of age-related pathologies^2,4,5^. Senescent cells can be identified by morphological changes, loss of their proliferative capacity, increased lysosomal senescence-associated β-galactosidase (SA-β-gal) activity and secretion of pro-inflammatory cytokines, generally referred to as senescence-associated secretory phenotype (SASP)^6,7^. In addition, senescent cells exhibit chromatin reorganization, and expression of p16^INK4A^, p21^CIP1^ and plasminogen activator type 1 (PAI-1)^2,8,9^. However, most senescence biomarkers are difficult to quantify or not exclusively expressed in senescent cells. Consequently, to study the role of senescence in tissue ageing and regeneration, it is essential to identify additional markers that facilitate the detection and quantification of senescent cells *in vitro* and *in vivo*.

We and others previously reported that downregulation of lamin B1 is a novel biomarker for senescence: lamin B1 was downregulated in fibroblasts undergoing replicative- or oncogene-induced senescence *in vitro* ^10–13^, in fibroblasts derived from patients with Hutchinson-Gilford progeria, an accelerated ageing syndrome^14,15^, in senescent melanocytes of benign human nevi^4^ and in chronologically aged human skin^10^. Furthermore, lamin B1 levels were used to quantify senescence in the liver of irradiated mice^12^ and in the kidney of a fast ageing *Xpd* ^*TTD/TTD*^ mouse model^16^. Skin ageing is an inevitable consequence of life and accelerated by exposure to ultraviolet (UV) rays. Chronic UVB exposure results in increased SA-β-gal activity, impaired proliferation and altered expression of extracellular matrix components^17^. However, it remains difficult to quantify the impact of UV-exposure on senescence in skin. Here we show that loss of lamin B1 can be used to detect and quantify senescence in primary human keratinocytes upon increasing doses of UV-exposure *in vitro*. To demonstrate the physiological relevance of these findings *in vivo*, we used a mouse model to quantify the accumulation and clearance of senescent cells after UV exposure and upon skin regeneration, respectively.

## Results

### Senescence and lamin B1 loss in UV-irradiated keratinocytes

**Figure 1:**
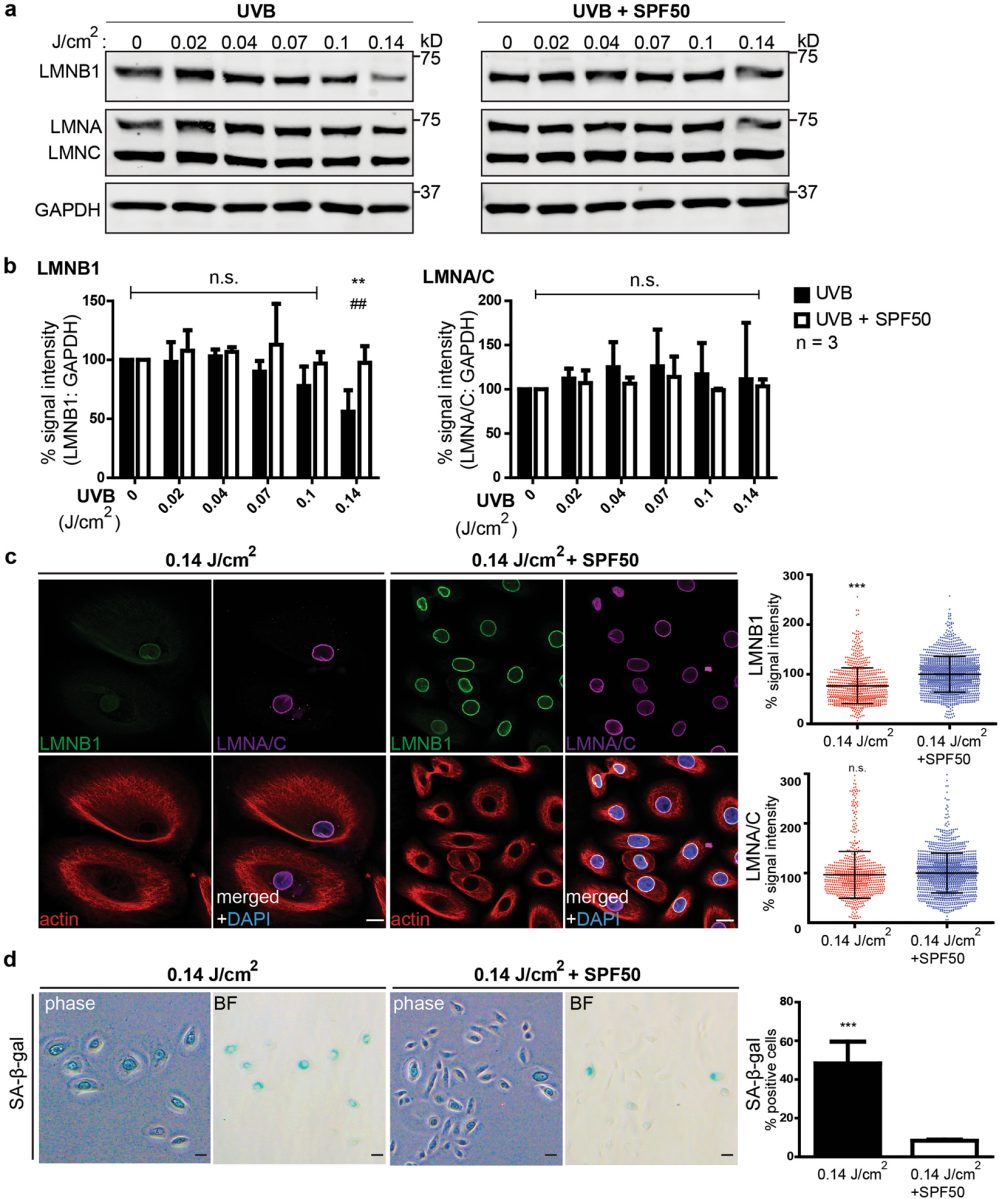
Lamin B1 loss and senescence in UV-exposed keratinocytes *in vitro*. (**a**) Representative western blot showing lamin B1 (LMNB1), lamin A (LMNA) and lamin C (LMNC) levels in primary keratinocytes exposed to increasing doses of UVB, in the presence of a plate covered in non-SPF base cream (left panels) or SPF50+ sunscreen (right panels). (**b**) Quantification of LMNB1 and LMNA/C intensity (normalized to GAPDH and no UV-treated controls; n = 3 different keratinocyte lines; n.s.; non-significant). (**c**) Representative immunofluorescence images of LMNB1 (green) and LMNA/C (purple) expression in 0.14 J/cm^2^ UVB-treated and SPF50+ sunscreen-protected keratinocytes (actin, red; DAPI, blue). Bars, 10 µm. Right panels: Quantification of LMNB1 and LMNA/C levels. n = 3 different keratinocyte lines; ***P < 0.001 n.s.; non-significant. (**d**) Representative phase contrast (phase) and bright-field (BF) images of senescence-associated beta-galactosidase (SA-β-gal) staining in 0.14 J/cm^2^ UVB-treated and SPF50 + sunscreen-protected keratinocytes. Bars, 20 µm. Right panel: Quantification of SA-β-gal-positive cells. n = 3 different keratinocyte lines; all graphs show mean ± SD, ***P < 0.001.

To investigate whether lamin B1 levels can be used to quantify senescence during photoageing, we exposed primary human dermal keratinocytes from 4 different donors to increasing doses of UVB irradiation and quantified lamin B1 protein levels. UVB lamp exposures ranged from 0–120 seconds (0–0.14 J/cm^2^), corresponding to midday sunlight exposures of ~0–14 minutes (Supplementary Fig. 1). We observed an inverse correlation between UV-exposure and lamin B1 levels, with a significant loss of lamin B1 in keratinocytes exposed to 0.14 J/cm^2^ UVB. This loss was specific to lamin B1, as other nuclear lamina components lamins A and C remained unaltered (Fig. 1a–c, Supplementary Fig. 2). Immunofluorescence analysis confirmed that UV-exposed keratinocytes exhibited lower lamin B1 expression (Fig. 1c), became enlarged (Fig. 1c,d) and expressed SA-β-gal activity (Fig. 1d), which are characteristic of senescent cells. Importantly, application of SPF50+ sunscreen prior to UV-exposure prevented lamin B1 loss and expression of SA-β-gal activity (Fig. 1a–d, Supplementary Fig. 2). Lastly, concomitant with the loss of lamin B1 protein levels, we observed a significant reduction in lamin B1 transcript (*LMNB1*) levels starting from 0.04 J/cm^2^ UVB (Supplementary Fig. 3). In contrast, upregulation of *CDKN2A* (p16^INK4a^), *CDKN1A* (p21^CIP1^) and *SERPINE1* (PAI-1) only occurred at exposures higher than 0.1 J/cm^2^ UVB, while lamin A/C (*LMNA*) transcript levels did not change significantly (Supplementary Fig. 3). These results demonstrate that lamin B1 loss is an early and sensitive marker to quantify UV-induced senescence *in vitro*.

### Lamin B1 levels are reduced in mouse skin upon chronic UV-exposure

**Figure 2:**
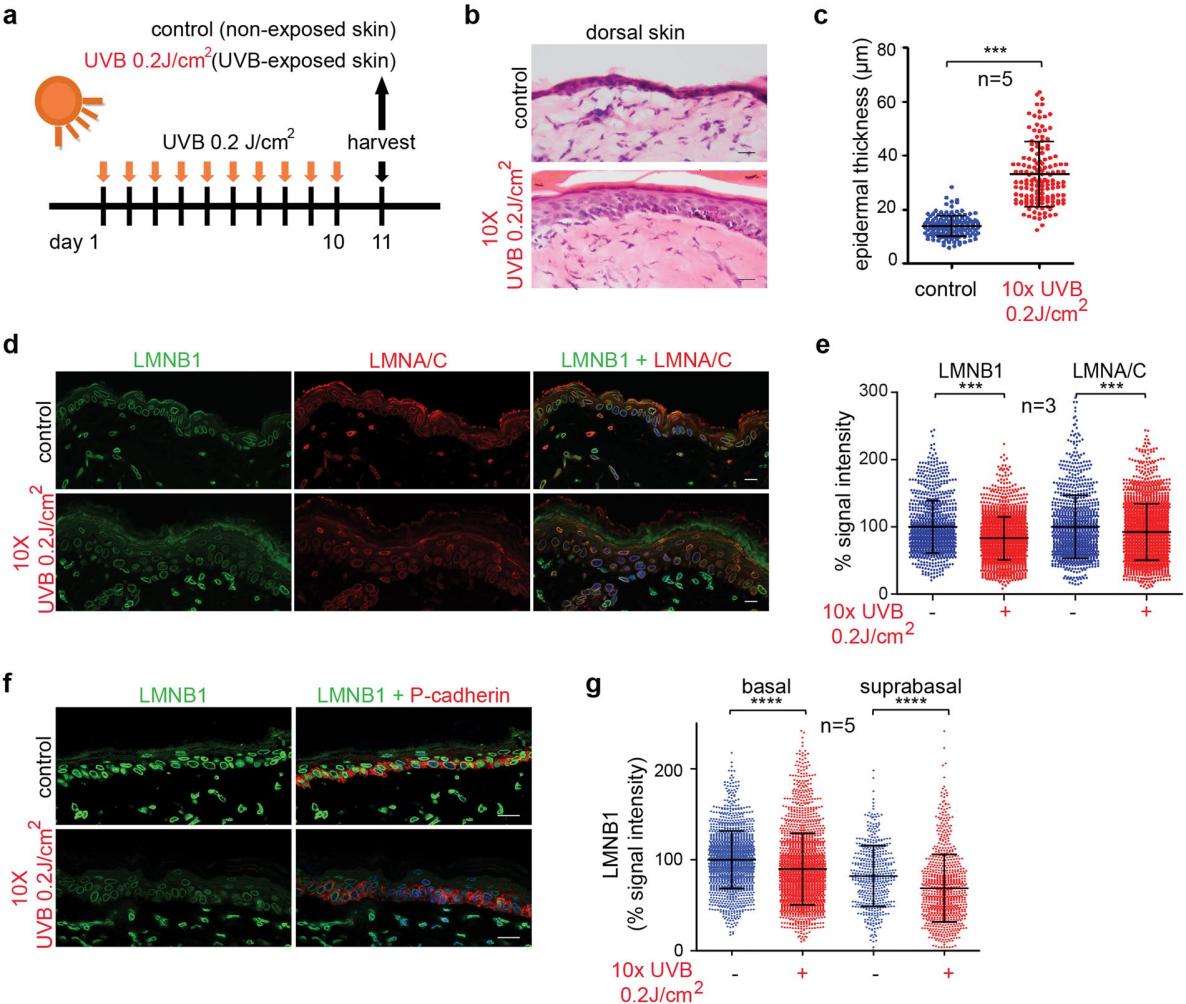
Chronic UV-exposure results in lamin B1 reduction in mouse skin. (**a**) Experimental set-up. (**b**) H&E staining of UV-exposed versus control skin. (**c**) Quantification of epidermal thickness (n = 5). (**d**) Immunofluorescence analysis of lamin B1 and lamin A/C expression (LMNB1; green, LMNA/C; red). Bars, 20 µm. (**e**) Quantification of LMNB1 and LMNA/C levels throughout the epidermis, normalized to non-irradiated controls. n = 3, ***P < 0.001. (**f**) Immunofluorescence analysis of lamin B1 expression (LMNB1; green, P-cadherin; red). Bars, 20 µm. (**g**) Quantification of LMNB1 in basal and suprabasal layers, normalized to LMNB1 intensity of non-irradiated basal cells; n = 5, all graphs show mean ± SD, ****P < 0.0001).

To determine the physiological relevance of these findings *in vivo*, we irradiated dorsal skin of mice with 0.2 J/cm^2^ UVB for 10 consecutive days (Fig. 2a), as described previously^18^. Histological and immunofluorescence analysis revealed an increase in epidermal thickness (Fig. 2b–d), accumulation of DNA damage (by γ-H2A-X staining; Supplementary Fig. 4a) and loss of lamin B1 (~17.6%) throughout the epidermis upon UV irradiation (Fig. 2d,e). Concomitant with the loss of lamin B1 protein, we observed an increase in the mRNA of well-characterized senescence markers including PAI-1 (*Serpine1*), p16 and p19 (*Cdkn2A*). Exposure to UVB did not alter *Lmna* mRNA levels in UV-exposed mouse epidermis (Supplementary Fig. S5). However, we observed a slight reduction of lamin A/C protein levels (~7.7%) (Fig. 2d,e).

To determine whether senescent cells accumulate in specific skin compartments, we probed skin sections with lamin B1 and P-cadherin, a cell adhesion molecule that exclusively marks the basal layer within the epidermis (Fig. 2f) and quantified lamin B1 levels in single cells from the basal or suprabasal layer (Fig. 2g). UV-irradiated skin epidermis showed a 10.2% and 16.2% decrease in lamin B1 intensity in basal and suprabasal cells, respectively (Fig. 2g). In contrast to the epidermal layer, we did not observe a reduction in lamin B1 in the underlying dermal fibroblasts (Fig. 2f).

### Lamin B1 levels are restored upon skin regeneration

**Figure 3:**
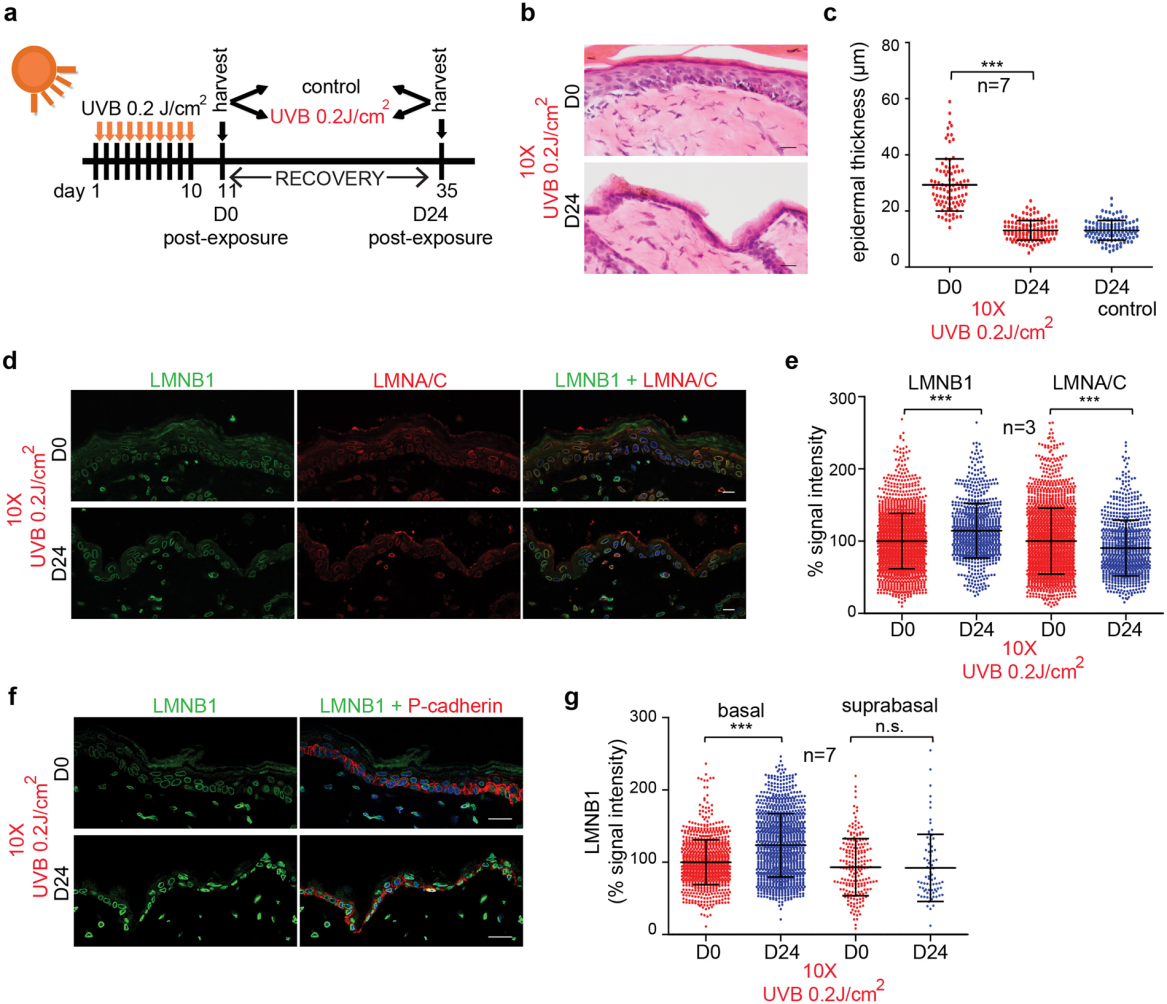
Restoration of lamin B1 levels upon skin regeneration. (**a**) Experimental set-up. (**b**) H&E staining of skin post-irradiation (D0) and after recovery (D24). (**c**) Quantification of epidermal thickness (n = 7). (**d**) Immunofluorescence analysis of LMNB1 (green) and LMNA/C (red) expression in D0 versus D24 skin. Bars, 20 µm. (**e**) Quantification of LMNB1 and LMNA/C at D0 versus D24; normalized to intensity of D0; n = 3; ***P < 0.001. (**f**) Immunofluorescence analysis of LMNB1 (green) and P-cadherin (red) expression in D0 versus D24 skin. Bars, 20 µm. (**g**) Quantification of LMNB1 in basal and suprabasal layers D0 versus D24; normalized to LMNB1 intensity of D0 basal cells; n = 7; all graphs show mean ± SD, ***P < 0.001, n.s.; non-significant.

To investigate whether lamin B1 levels can be used to study skin regeneration after UV-exposure, we UV-treated mice for 10 consecutive days, allowed them to recover for 24 days and then analyzed lamin B1 levels (Fig. 3a). Strikingly, the epidermal layer thinned back to normal thickness (Fig. 3b,c), exhibited no DNA damage (Supplementary Fig. 4b) and lamin B1 levels were restored upon regeneration (~14.5%). In contrast, lamin A/C levels decreased further (~9.6%) (Fig. 3d,e). To test whether we could detect skin regeneration specifically within the basal layer of the epidermis, we co-stained skin sections with lamin B1 and P-cadherin (Fig. 3f). Lamin B1 levels were restored in the basal layer whereas cells residing in the suprabasal layer still exhibited low lamin B1 levels (Fig. 3f,g). Since skin regeneration originates within the basal layer, we speculate that this results in complete restoration of lamin B1 levels within this compartment. However, as keratinocytes migrate apically, the suprabasal layer may still contain senescent cells (with low levels of lamin B1) which were originally located in the basal layer.

## Discussion

We and others previously showed that the loss of lamin B1 is a robust marker to detect senescent cells^4,10,12,13,16^. Mechanistically, loss of lamin B1 in senescent cells is regulated at both transcript and protein levels. *LMNB1* transcripts decrease by ~20-fold in senescent cell types^10,12,13^ and are targeted by miR-23a in senescent cells, as well as during postnatal development of the central nervous system^10,19^. In addition, autophagy may contribute to the rapid degradation of lamin B1 protein in senescent cells^20^. While loss of lamin B1 plays a key role in senescence-associated changes in chromatin organization^21–23^, the upstream pathways that initiate lamin B1 reduction remain unclear. p63, a key mediator in skin morphogenesis and homeostasis, may play a role in regulating lamin B1: epidermal levels of p63 decrease during chronological ageing and upon exposure to UVB^24–26^, and p63 depletion triggers cellular senescence, chromatin remodeling and loss of lamin B1 in mouse skin^27,28^. Although the causality and temporal chain of events that lead to lamin B1 loss in senescent cells remains to be determined, these studies suggest that the regulation of lamin B1 is likely to be orchestrated by multiple pathways, and at the level of transcription and protein stability.

Here we demonstrate that lamin B1 can be used to quantify the impact of environmental insults, such as UV-exposure on primary human keratinocytes *in vitro*, as well as on skin ageing and regeneration *in vivo*. By comparing the transcriptional profile of *LMNB1* and other senescence markers (*CDKN2A* (p16^INK4a^), *CDKN1A* (p21^CIP1^) and *SERPINE1* (PAI-1)) upon different UVB exposures, we observed a significant reduction in *LMNB1* transcript levels at a low dose of 0.04 J/cm^2^ UVB whereas PAI-1, p21 and p16 upregulation only occurred at UVB doses higher than 0.1 J/cm^2^ (Supplementary Fig. 3). Based on these results, we speculate that loss of *LMNB1* is an earlier and possibly more sensitive marker for senescence. In agreement with our findings, Freund *et al*. previously demonstrated that loss of lamin B1 preceded the activation of SA-β-gal and SASP^12^.

UV-exposed skin is histologically characterized by a prominent thickening of the epidermis^29,30^. Increased epidermal thickening has previously been observed in *Lmna* knockout mice, and in mice expressing progerin, a mutated form of lamin A that is associated with the premature ageing syndrome progeria^31–33^. To address whether lamin A/C levels correlated with senescence or epidermal thickening, we measured lamin A/C levels before and after UV-exposure, as well as upon skin regeneration. We did not observe any consistent correlation between lamin A/C levels and epidermal thickening/thinning, suggesting that changes in epidermal thickness cannot be attributed to changes in lamin A/C. In addition, the consequences of UV-exposure on lamin A/C differed between our *in vitro* and *in vivo* experiments: While a single dose of UV had no effect on lamin A/C levels *in vitro*, chronic UV exposure *in vivo* led to a slight decrease in lamin A/C. Moreover, lamin A/C levels decreased further upon skin regeneration. This apparent lack of correlation might not be unexpected as the regulation of lamin A/C is orchestrated by a more complex set of parameters: lamin A/C expression varies within different compartment of the skin^34,35^, in different cell types and tissues^36,37^, during cellular differentiation^38^ and by different stimuli that trigger senescence^12^. Thus, in contrast to lamin B1, lamin A/C is not a reliable marker to identify senescent cells nor a characteristic of ageing skin^10,12^.

In conclusion, we demonstrate that lamin B1 can be used to quantify the impact of environmental insults, such as UV-exposure, on skin ageing and regeneration *in vivo*. Importantly, by co-staining with cell type specific markers, lamin B1 allows the identification of specific cell types that have undergone senescence in different tissue compartments or organelles. This convenient and specific identification of senescent cells facilitates (1) the detection of senescent cells in various age-related pathologies, including chronic inflammation, (2) serves as a diagnostic tool to distinguish senescent from proliferating cells in pre-neoplastic lesions, and (3) provides a simple and quantifiable method to study the role of senescence in tissue ageing and regeneration in response to different environmental conditions, such as pollution or diet.

## Methods

### Keratinocyte cell culture and UV irradiation

Experimental procedures and methods were approved and performed in accordance to the guidelines of the Institute of Medical Biology, Institutional Biosafety Committee (IMB IBC). Primary human dermal keratinocytes were obtained with informed consent from 4 different donors. Tissue collection and cell banking at the Institute of Medical Biology was approved by SingHealth Institutional Review Board. Experiments were approved by the National University of Singapore Institutional Review Board. Keratinocytes were maintained under standard conditions (37 °C, 5% CO_2_) in DermaLife K Keratinocyte Medium Complete Kit (Lifeline Cell Technology) supplemented with 50 U/ml penicillin and streptomycin (Invitrogen). For UV irradiation experiments, primary keratinocytes were seeded on 6 cm dishes, incubated for 24 hours and washed once in phosphate buffered saline pH 7.4 before exposed to UV irradiation using a UV lamp (Ultra-Vitalux 300, Osram). UVB flux was measured by a UVB digital radiometer (Model HD2302.0, Delta Ohm). Either non-SPF base cream or SPF50+ sunscreen cream (Neutrogena Ultra Sheer Pure-Mild) was applied on UV-transparent plastic lids (HelioScreen, HD6, Labsphere), left to absorb for 15 minutes and placed over the 6 cm dish during UV irradiation. After exposure, PBS was replaced by keratinocyte medium and cells were incubated for another 3 days.

### Western blotting

Primary keratinocytes were harvested 3 days post UV irradiation. For western blot analysis, whole cell lysates were isolated using Complete Lysis-M solution (Roche). Protein concentration was determined with BCA protein assay kit (Pierce, Thermo Scientific). Lysates were subjected to SDS-PAGE, transferred to nitrocellulose membrane and blocked with Odyssey Blocking Buffer (Li-Cor Biosciences). Membranes were incubated with primary antibodies (lamin B1 (YenZym), lamin A/C (Millipore; MAB3211), GAPDH (Sigma; G9545)) overnight at 4 °C, washed in PBS and incubated in Odyssey IR Dye secondary antibodies. Odyssey Infrared Imaging System (Li-Cor Biosciences) was used for signal visualization and quantification. Full length gels are shown in Supplementary Figure 6.

### Immunofluorescence microscopy

Primary human keratinocytes were seeded on glass cover slips in 6 cm dishes and incubated for 24 hours prior to UV irradiation. At 3 days post UV irradiation, cover slips were fixed with 2% paraformaldehyde for 15 mins, followed by incubation with primary antibodies lamin B1 (YenZym), lamin A/C (Santa Cruz, SC-6215), actin (Sigma, A5441) for 1 hour and secondary antibodies and DAPI for 30 mins. Slides were mounted in Prolong-Gold Anti-fade reagent (Invitrogen) and immunofluorescence images acquired on a Olympus FV1000 inverted confocal laser scanning microscope.

### SA-β-gal analysis

Detection of SA-β-gal activity was performed as previously described^6,39^. Briefly, primary keratinocytes were fixed in 2% paraformaldehyde and 0.2% glutaraldehyde in PBS for 5 mins at room temperature, washed in PBS and stained in X-gal (5-bromo-4-chloro-3-indolyl-β-D-galactopyranoside) for 6 hours. Quantification was performed on 300–500 cells from each of 3 different primary keratinocyte lines. SA-β-gal-positive cells are represented as percentage of total cell number.

### Real-Time Quantitative Reverse Transcription PCR

Total RNA from primary human keratinocytes was extracted using Macherey-Nagel NucleoSpin RNA purification kit according to manufacturer’s instructions. The quantity and quality of total RNA was determined using a NanoDrop spectrophotometer (Thermo Scientific). 1.0 μg of RNA was reverse transcribed to cDNA using iScript Reverse Transcription Supermix (Biorad). qRT-PCR was performed on 7500 Fast Real-Time PCR system (Applied Biosystems) with TaqMan® Fast Universal PCR Master Mix (Applied Biosystems) or Fast SYBR® Green Master Mix (Applied Biosystems).

To isolate RNA from mouse epidermis, mouse dorsal skin was removed and washed in ice-cold 70% ethanol for 1 minute, followed by 1–2 minutes in ice-cold PBS. To separate dermis and epidermis, dorsal skin was placed dermis side up and muscles and fat were removed. Whole skin was then incubated overnight at 4 °C in Hanks Balanced Salt Solution (HBSS) containing 0.25% trypsin. Epidermal cells were scraped off, incubated in 0.25% trypsin solution for 15 mins at 37 °C and resuspended by intermittent pipetting. Cells were filtered through a 70 µm sterile filter, centrifuged at 1000 rpm for 8 minutes, washed twice with cold PBS^40^ and total RNA was extracted using Qiagen RNeasy purification kit. 1.0 μg of RNA was reverse transcribed to cDNA using iScript Reverse Transcription Supermix (Biorad). qRT-PCR was performed on a Roche LightCycler® 480 with LightCycler ® 480 SYBR Green I Master (Roche).

### Mouse husbandry and UV irradiation

All mice experiments were approved by the A*STAR Biological Resource Centre Institutional Animal Care and Use Committee (IACUC) review board. Mice were kept in cages with 12 hour light periods at the AAALAC-accredited animal facility in the Biological Resource Centre, A*STAR, Singapore in compliance with the National Advisory Committee for Laboratory Animal Research (NACLAR) guidelines. Mice were supplied with Altromin standard diet pellets and drinking water *ad libitum*. For UV irradiation, the backs of 7-weeks old C57BL6 littermate mice were shaved 3 days before the first UV dose. Subsequently, mice were irradiated with a daily dose of 0.2 J/cm^2^ UVB for 10 consecutive days as previously described^18^ using a UV lamp (Ultra-Vitalux, Osram). UVB flux was measured using a UVB digital radiometer (Model HD2302.0, Delta Ohm). One day after the last irradiation, mice were sacrificed by CO_2_ euthanasia and their dorsal skin was harvested. For the skin recovery studies, mice were UV-irradiated for 10 consecutive days (0.2 J/cm^2^ UVB), and dorsal skin was harvested 24 days later. For all experiments, dorsal skin shielded from UV irradiation was used as an isogenic non-irradiated control.

### Histological analysis and immunofluorescence microscopy

Mouse dorsal skin was excised and immediately embedded in OCT compound. For histological studies, skin tissue sections (~7 µm) were stained in hematoxylin and eosin (H&E) and imaged using Zeiss Axio Imager Upright Microscope. To measure the skin epidermal thickness, the average of at least 30 widths at random locations along the length of epidermis was measured (Adobe Photoshop CC). Results were compiled from at least 3 biological replicates for all treatment groups.

For immunofluorescence, skin sections were fixed in 4% paraformaldehyde (PFA), permeabilized and blocked with 3% normal donkey serum containing 0.1% SDS and 0.1% Triton X-100, then incubated with primary antibodies overnight at 4 °C. Slides were washed in PBS and incubated in secondary antibodies and DAPI for 1 hour, before mounted in Prolong-Gold Anti-fade reagent (Invitrogen). Immunofluorescence images were taken on a Olympus FV1000 upright confocal laser scanning microscope. The following primary antibodies were used: lamin B1 (YenZym), P-cadherin (R&D Systems; AF761), γ-H2A-X (Ser139; Millipore; 05–636) and lamin A/C (Santa Cruz; SC-6215).

### Quantification of lamin B1 and lamin A/C levels using CellProfiler

To measure lamin B1 and lamin A/C levels in mouse skin, at least 10 immunofluorescence images were captured at random along the length of dorsal skin epidermis. The number of biological replicates of each treatment group is indicated in respective figure legends. For quantification, animal sections or immunofluorescence microscopy images were numbered and analyzed using CellProfiler without prior knowledge of treatment group.

### Statistical analysis

All data and statistical analysis were performed using GraphPad Prism software. Results are shown as mean ± SD in text and figures. Data were analyzed using Student’s t-test or two-way ANOVA, followed by post hoc Bonferoni method for multiple comparisons as appropriate. (*P < 0.05, **P < 0.01, ***P < 0.001). P < 0.05 values were considered significant.

### Data availability

All data generated or analysed during this study are included in this published article and its supplementary information files.

## Electronic supplementary material

Supplementary Information
