# Supplementary material for: Loss of lamin B1 is a biomarker to quantify cellular senescence in photoaged skin
Source: Sci Rep. 2017 Nov 15;7:15678. doi: 10.1038/s41598-017-15901-9 (PMC5688158; doi:10.1038/s41598-017-15901-9)

Loss of lamin B1 is a biomarker to quantify cellular senescence in photoaged skin

Audrey Shimei Wang[1#], Peh Fern Ong[1#], Alexandre Chojnowski[2], Carlos Clavel[3*] and Oliver Dreesen[1*]

[1]Cell Ageing, Institute of Medical Biology, [2] Developmental and Regenerative Biology, [3]Hair & Pigment Development, Institute of Medical Biology, 8A Biomedical Grove, #06-06 Immunos, 138648 Singapore

SUPPLEMENTARY INFORMATION

Supplementary information contains

Supplementary Figure legends & Supplementary Figures 1-6

Supplementary figure legends

Supplementary Figure 1: UVB quantification: UV lamp as compared to mid-day sunlight exposure. UVB flux in J/cm^2 of the Ultra-Vitalux 300 lamp (grey bars) compared to mid-day sunlight (white bars) at different exposure times (0-120 seconds); n=3, graphs show mean ± standard deviation (SD).

Supplementary Figure 2: Lamin B1 reduction in UV-exposed primary keratinocytes *in vitro*. Western blots showing lamin B1 (LMNB1), lamin A (LMNA) and lamin C (LMNC) levels in keratinocytes from 3 different donors. UVB exposures are indicated in J/cm^2. Cells were UV-treated in the presence of a plate covered in moisturizer (left panels) or SPF50+ sunscreen (right panels).

Supplementary Figure 3: Reduction of lamin B1 transcript (*LMNB1*) levels in UV-exposed keratinocytes. Transcript levels of *LMNB1*, *LMNA* (lamin A/C), *CDKN2A* (p16^{INK4A}), *CDKN1A* (p21^{CIP1}) and *SERPINE1* (PAI-1) in UV-irradiated keratinocytes were normalized to non-UV irradiated controls (n=3). Graphs show mean ± SD. n.s.; non-significant, */# P<0.05, **/## P<0.05, ***/### P<0.001. Statistical comparison of transcript levels of UV-irradiated versus non-irradiated keratinocytes. # Statistical comparison of transcript levels of UV-irradiated versus SPF50+ protected keratinocytes at the same UVB dose.

Supplementary Figure 4: Transcript levels of senescence markers and *Lmna* in UV-exposed versus control mouse epidermis. Transcript levels of *Lmna*, *Serpine1* (PAI-1), *Cdk2A* (p16 and p19ARF) and *Cdkn1a* (p21) of UV-irradiated (10x UVB 0.2

J/cm^2) mouse skin epidermis, normalized to non-irradiated controls (n=3). Graphs show mean ± SD. n.s.; non-significant, $^*P<0.05$.

Supplementary Figure 5: **UV-exposed mouse skin exhibits DNA damage.** (**a**) Increased γ-H2A-X staining in epidermal cells with reduced LMNB1 levels upon 0.2 J/cm^2 UVB exposure (white dotted line separates epidermis and dermis). (**b**) Lamin B1 (LMNB1) and γ-H2A-X staining on skin 24 days post UVB exposure (D24) compared to D0 (white dotted line separates epidermis and dermis). Bars, 20 µm.

Supplementary Figure 6: Full length western blot / gels displayed in Figure 1a (main text) and Supplementary Figure 2.

Supplementary Figure 1

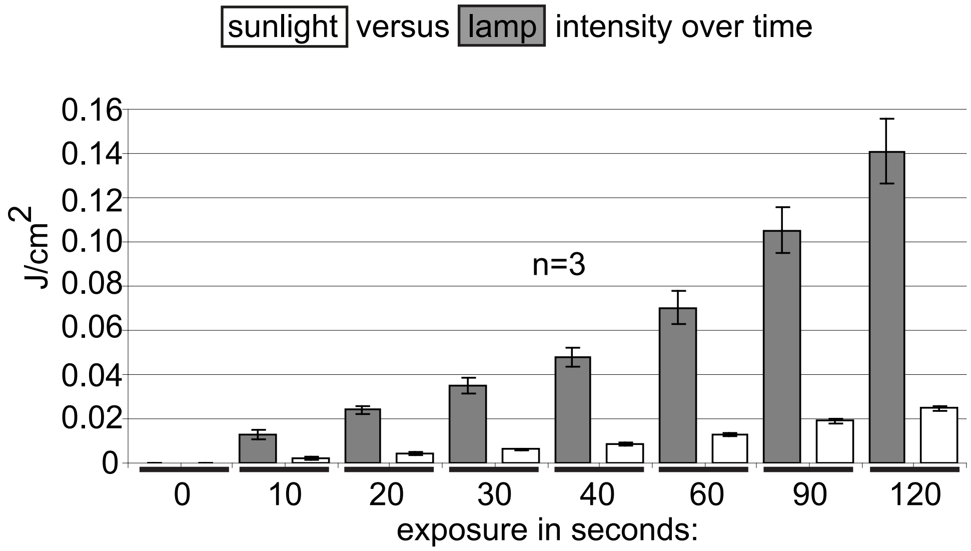

Supplementary Figure 2

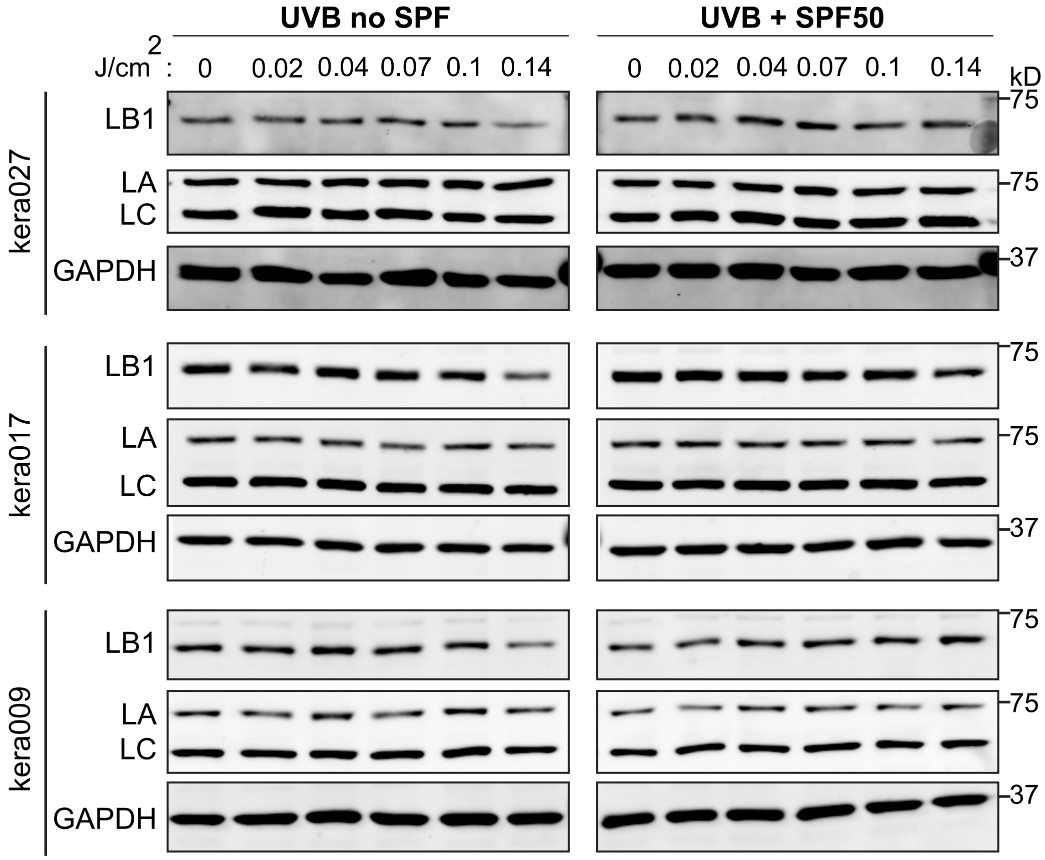

Supplementary Figure 3

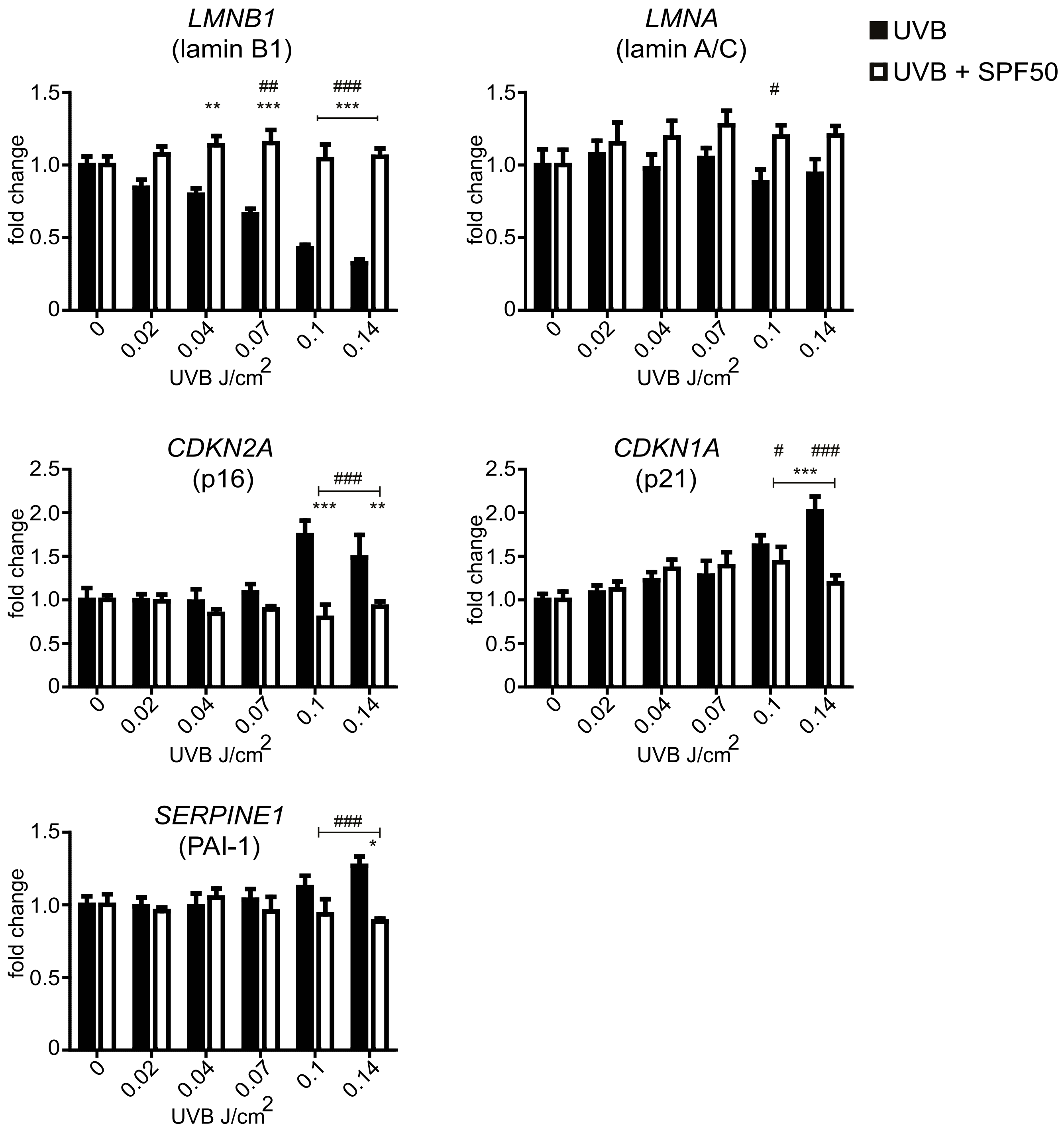

Supplementary Figure 4

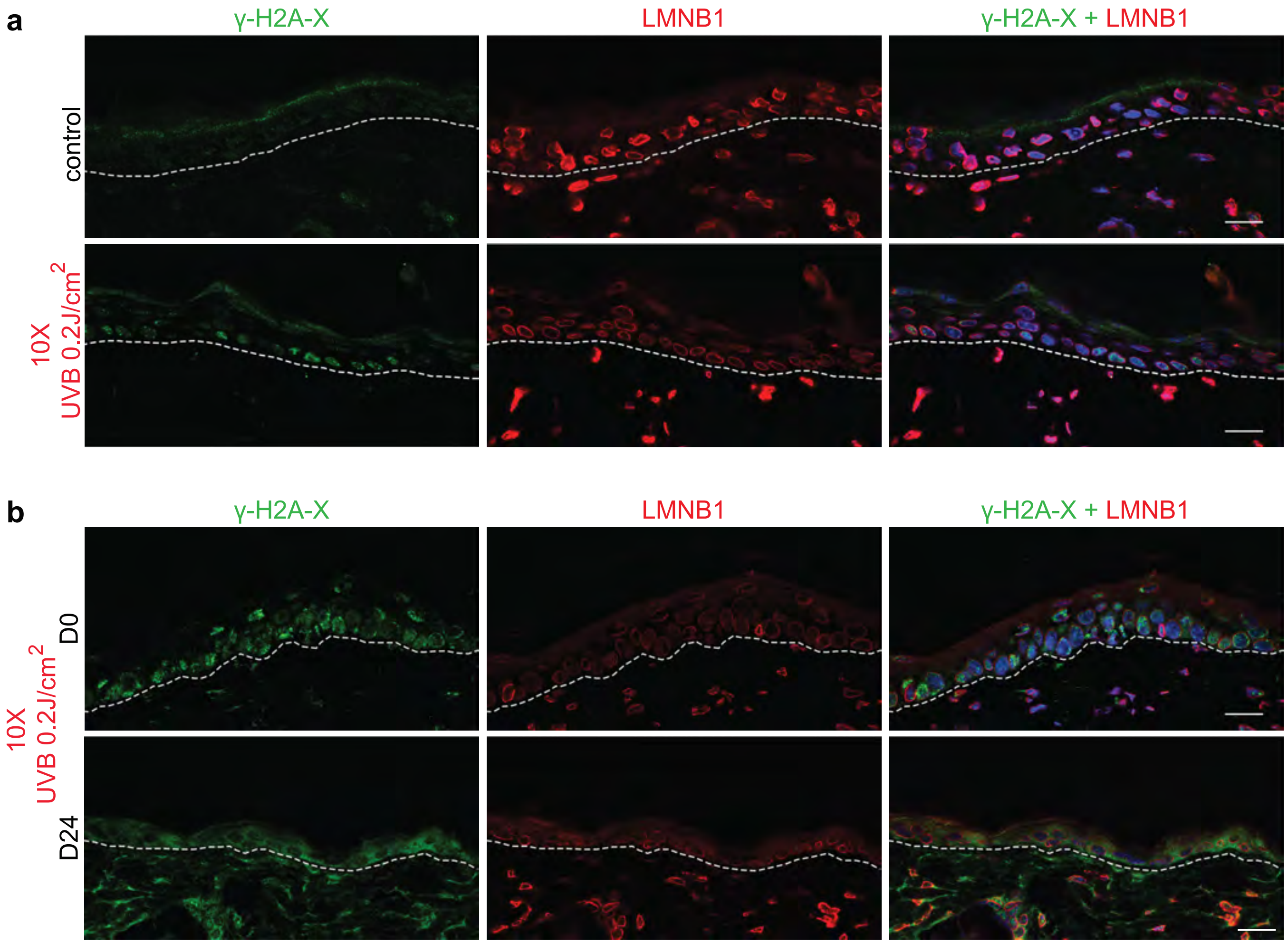

Supplementary Figure 5

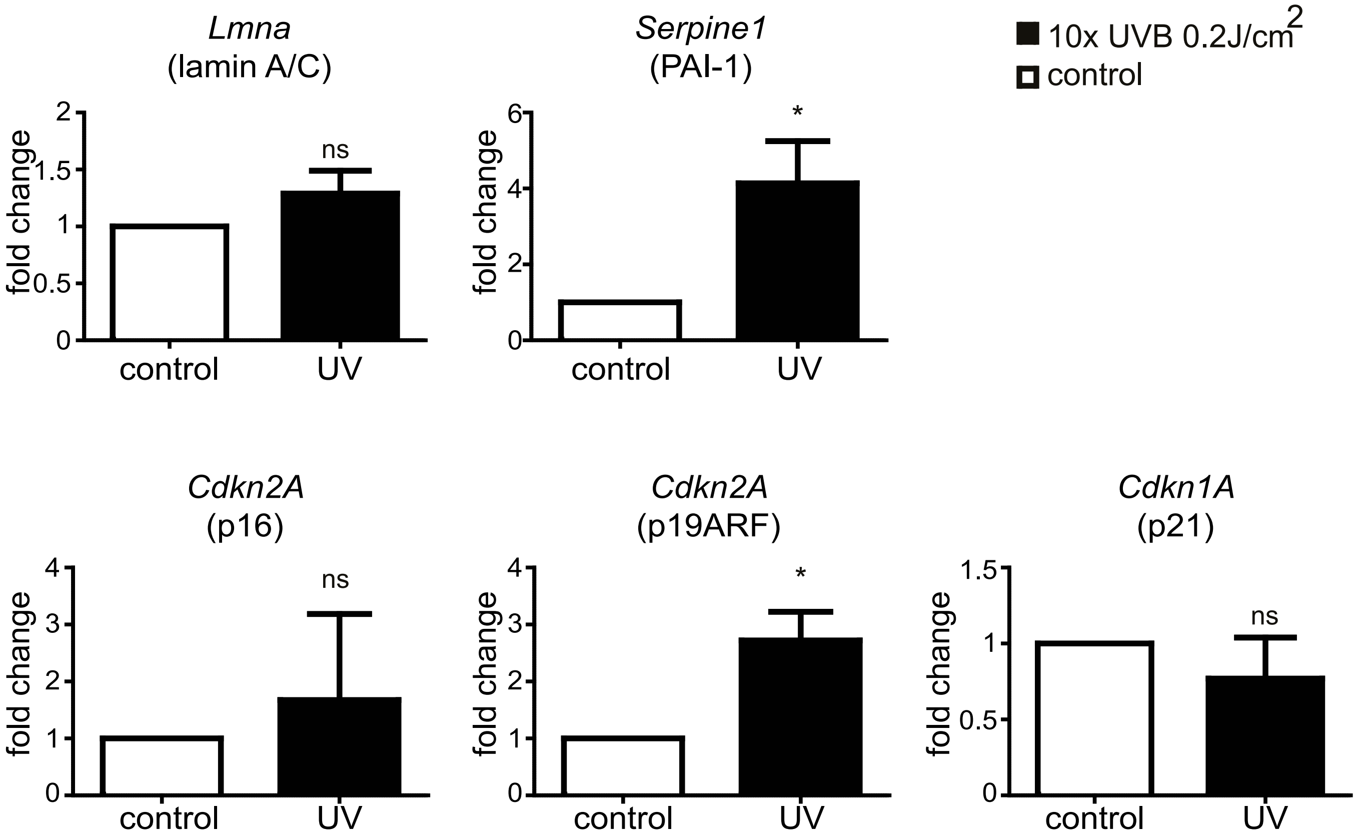

Supplementary Figure 6

full length gel of Western blot panels in Figure 1a

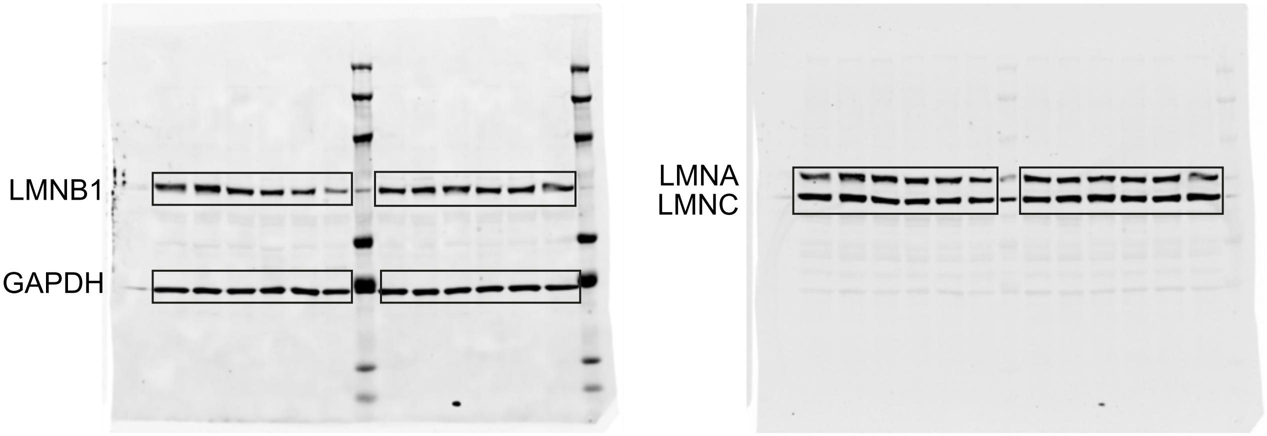

full length gel of Western blot panels in Supplementary Figure 2 keratinocyte line 1

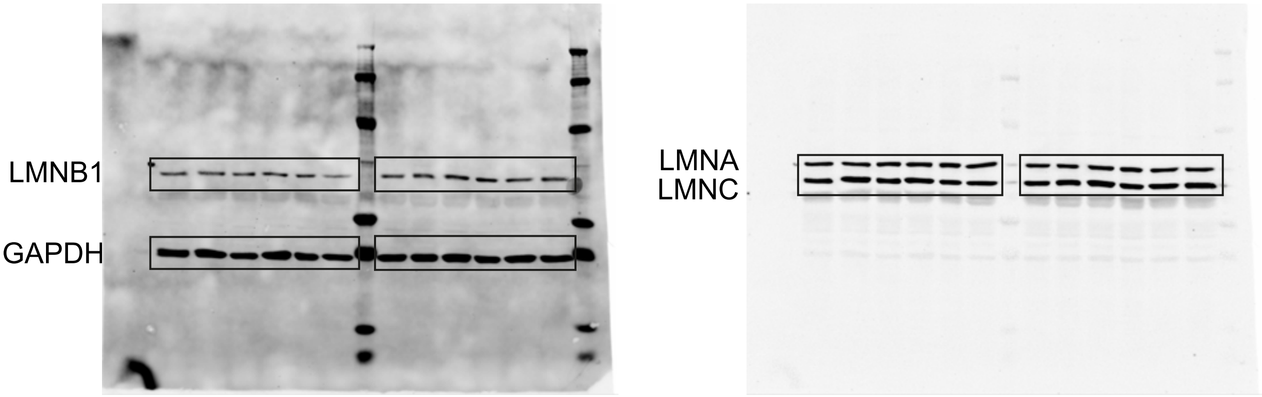

full length gel of Western blot panels in Supplementary Figure 2 keratinocyte line 2

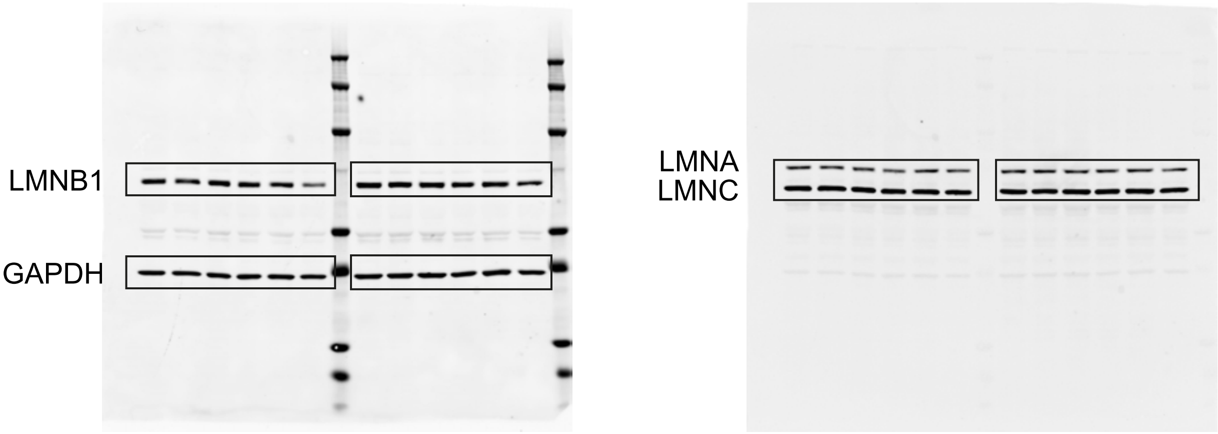

full length gel of Western blot panels in Supplementary Figure 2 keratinocyte line 3

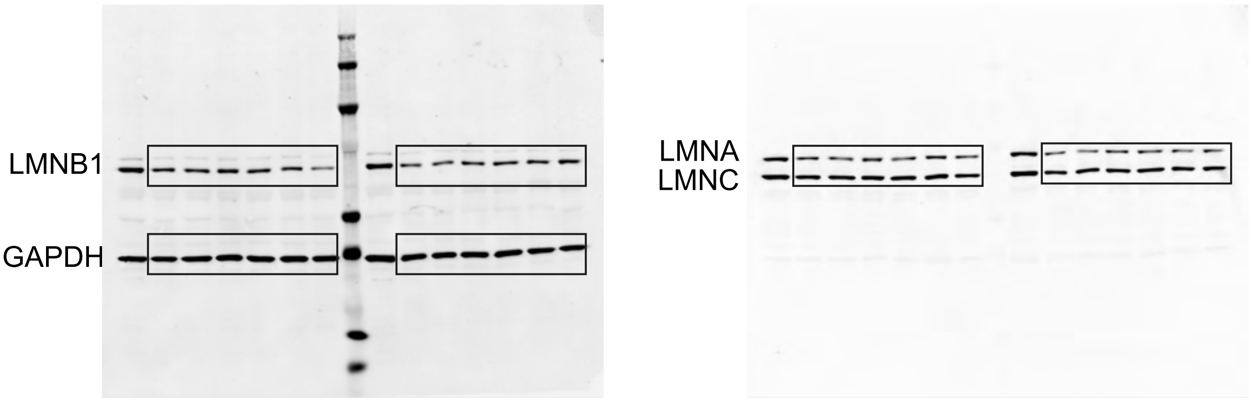

Supplement: Supplementary file 1 — Supplementary Information [file 41598_2017_15901_MOESM1_ESM.pdf]
